# Safety and Efficacy of Tien-Hsien Liquid Practical in Patients with Refractory Metastatic Breast Cancer: A Randomized, Double-Blind, Placebo-Controlled, Parallel-Group, Phase IIa Trial

**DOI:** 10.1155/2012/803239

**Published:** 2012-04-01

**Authors:** Wen-Hung Kuo, Chien-An Yao, Chih Hui Lin, King-Jen Chang

**Affiliations:** ^1^Department of General Surgery, National Taiwan University Hospital, Taipi, Taiwan; ^2^Department of Family Medicine, National Taiwan University Hospital, Taipi, Taiwan; ^3^Gerent Biotech Co., Ltd., Taichung, Taiwan; ^4^Department of Surgery, Cheng Ching General Hospital, Taichung, Taiwan

## Abstract

To evaluate the safety and efficacy of Tien-Hsien Liquid Practical (THL-P), a Chinese herbal mixture, in patients with refractory metastatic breast cancer, we performed a randomized, double-blind, placebo-controlled, parallel-group, phase IIa pilot trial. Patients were randomly assigned to either receive THL-P or matching placebo and followed up every 4 weeks for 24 weeks. The primary endpoint was changes in the global health status/quality of life (GHS/QOL) scale. The secondary endpoints were changes in functional and symptom scales, immunomodulating effects, and adverse events. Sixty-three patients were enrolled between June 2009 and June 2011. The intent-to-treat population included 28 patients in the THL-P group and 11 patients in the placebo group. Compared to the placebo group, the THL-P group had significant improvement from baseline to last visit in GHS/QOL (41.7 versus −33.3; *P* < 0.05), CD3, CD4/CD8, CD19, CD16+56 positive cells (*P* < 0.05), and higher levels of physical, role, emotional, and cognitive functioning, as well as decreased fatigue and systemic side effects. Treatment-related adverse events were mild constipation and localized itching, and no serious adverse events were reported. THL-P appears to be a safe alternative adjuvant treatment for patients with refractory metastatic breast cancer, as it effectively improves QOL and palliates cancer-related symptoms.

## 1. Introduction

Breast cancer, a leading cause of morbidity and mortality among women, was estimated to account for 23% (1.38 million) of new cancer cases and 14% (458,400) of cancer deaths worldwide in 2008 [1, 2]. In Taiwan, breast cancer was the most common cancer among women, with an incidence of 9,049 cases per 100,000 population, and the fourth leading cause of cancer-related deaths in 2008 [3]. Despite advances in the treatment of breast cancer, it is estimated that 30–50% of women initially diagnosed with earlier stages of breast cancer eventually develop metastatic disease [4]. Unfortunately, breast cancer has a poor prognosis following metastasis, and women with metastatic breast cancer have a limited survival of 18–24 months and a 5-year survival rate of approximately 20% [2, 5]. The progression to metastatic disease is further exacerbated by the increasing use of chemotherapy in early-stage breast cancer, which has led to a corresponding increase in the number of metastatic breast cancer cases refractory to conventional treatments [4].

The currently available conventional treatment options for metastatic breast cancer include cytotoxic chemotherapy, endocrine/hormonal therapy, targeted biological therapy, radiation therapy, bisphosphonates, or combinations of these, and surgery in some cases [2, 4, 5]. However, there is no global consensus and guidelines for the treatment of metastatic breast cancer, and treatment is highly individualized and varies worldwide. Furthermore, due to the poor prognosis of metastatic breast cancer, the main goals of treatment are palliative rather than curative. Specifically, treatment is aimed at prolonging progression-free survival (PFS) and overall survival (OS), managing or reducing disease symptoms, and achieving the best quality of life (QOL) [4, 5]. However, the significant side effects, potential for the development of treatment resistance, and limited survival benefit associated with conventional treatments have prompted the use of complementary and alternative medicine (CAM) in patients with advanced malignancies [4, 6–8].

The use of CAM has been on the rise, especially among patients with life-threatening diseases, such as cancer [9]. In fact, it has been estimated that 48–98% of breast cancer patients use CAM, and the most commonly used CAM is herbal medicine [10–13]. There are numerous reasons cited by breast cancer patients for using CAM, including strengthening the immune system, increasing QOL, treating or preventing the recurrence of cancer, stabilizing current condition, alleviating cancer-related symptoms, assisting conventional treatments, relieving symptoms and stress associated with side effects of conventional treatments, providing a feeling of control over life, reducing stress and detoxification, and compensating for failed conventional medical treatments [10, 12, 13]. Although CAM is widely used by breast cancer patients, their safety and efficacy have been studied in very few randomized and controlled clinical trials [13].

Tien-Hsien Liquid Practical (THL-P), a Chinese herbal mixture, has been used as a CAM for over ten years. Recently, THL-P has been shown to have strong immunomodulating and anticancer effects via a number of preclinical *in vitro *and *in vivo *experiments [6, 7, 9, 14, 15]. Specifically, THL-P has been shown to modulate the antigen-stimulated proliferation response and cytokine production of T-lymphocytes, inhibit cell growth and induce apoptosis in various human cancer cell lines and possess antitumour, antiangiogenic, antimetastatic effects [6, 7, 9, 14, 15]. Based on these promising findings, we performed a randomized, double-blind, placebo-controlled, parallel-group, phase IIa pilot trial to evaluate the safety and efficacy profiles of THL-P in patients with metastatic breast cancer refractory to conventional treatments.

## 2. Methods

### 2.1. Study Design

This study was a randomized, double-blind, placebo-controlled, parallel-group, phase IIa pilot trial in patients with metastatic breast cancer refractory to conventional treatment modalities. The primary outcome was the changes from baseline to posttreatment evaluations in the global health status/quality of life (GHS/QOL) standardized scale, assessed by the self-administered European Organization for Research and Treatment of Cancer Quality of Life Questionnaire-Core 30 (EORTC-QLQ-C30). The secondary outcomes included changes in functional and symptom scales, as well as the single items, of the EORTC-QLC-Q30 and -Breast Cancer 23 (BR23), immunomodulating effects of treatment on lymphocytes, and treatment-related adverse events. The study was approved by the Institutional Review Board (IRB) of the National Taiwan University Hospital, and written informed consents were obtained from all patients prior to entering the trial.

### 2.2. Participants

Subjects were recruited at the National Taiwan University Hospital in Taipei, Taiwan between June 2009 and June 2011. The inclusion criteria were women, aged 20 to 80 years old, who had histologically or cytologically confirmed breast cancer with clinical evidence of progressive metastatic disease and any one of the following: (a) no satisfactory response after primary or salvage treatment (i.e., chemotherapy, radiotherapy, surgery, or other approved therapies, such as target therapy or immunotherapy) and (b) no intention of accepting additional conventional treatments. Subjects were included provided that they had adequate bone marrow function with an absolute neutrophil count ≥1000/*μ*L, hemoglobin count ≥8 g/dL, and platelet count >75,000/*μ*L, and liver and renal function with total serum bilirubin <3 mg/dL and serum creatinine <2 mg/dL, respectively. Subjects of childbearing potential had to agree to use medically accepted means of contraception during the participation of the study. Subjects must also have an estimated life expectancy of at least 4 weeks. All subjects had to give written informed consent to participate in the study.

Subjects were excluded if they have ever received radiotherapy, endocrine therapy, antineoplastic drugs, or hormonal agents as adjuvant treatment or therapy for metastatic breast cancer within 2 weeks prior to entering the study. Additional exclusion criteria included uncontrolled infections, history of autoimmune disease (i.e., lupus erythematosus, ankylosing spondylosis, scleroderma or multiple sclerosis), prior history of other malignancies, with the exception of skin basal cell carcinoma, within 3 years of study entry, or any other serious diseases or medical history considered by the investigator to place the subject at increased risk. Subjects with aspartate aminotransferase (AST) and alanine aminotransferase (ALT) levels above five times the upper limit of normal values, or if liver metastases were present, above ten times the upper limit of normal values, were excluded. Women who were lactating, pregnant, or planning to become pregnant were also excluded. Lastly, subjects were not eligible if they had participated in any other investigational study within 4 weeks of study entry.

Subjects were withdrawn from the study if any of the following criteria were met: (a) subject decided to withdraw her informed consent, (b) the investigator considered the subject to be no longer physically or psychologically capable of remaining in the study, (c) subject refused to proceed with critical measures for the study endpoints, defined as all variables required for the primary endpoint analysis, or (d) subject developed adverse effects that the investigator considered as warranting discontinuation of the study treatment.

### 2.3. Treatment Groups

The study group received 1 vial (20 mL) of THL-P oral solution 3 times per day for 24 weeks. THL-P is an aqueous preparation that consists of extracts from 14 Chinese medicinal herbs, 11 of which are active ingredients and the remaining three are flavoring ingredients. The active ingredients of THL-P oral solution (Sheng Foong Co., Ltd., I-Lan County, Taiwan) are *Atractylodes macrocephala *(250 mg/mL)*, Astragalus membranaceus *(330 mg/mL)*, Taraxacum mongolicum *(500 mg/mL)*, Poria cocos *(330 mg/mL)*, Ligusticum chuanxiong* (250 mg/mL)*, Ligustrum lucidum* (250 mg/mL)*, Codonopsis pilosula* (250 mg/mL)*, Glycyrrhiza uralensis *(160 mg/mL)*, Hedyotis diffusa* (330 mg/mL)*, Pseudostellaria heterophylla *(250 mg/mL), and* Viola philippica* (160 mg/mL). The compositions, as well as the pharmacological and immunological effects, of these ingredients, have been previously described [14, 15]. The control group received 1 vial (20 mL) of a matched placebo 3 times per day for 24 weeks. The matched placebo consisted of food-grade flavoring ingredients, which ensured a similar appearance, taste, and odor to the THL-P oral solution, and was dispensed in a similar opaque plastic bottle.

 The investigators attempted to minimize the use of concomitant treatments in each subject throughout the study. If concomitant treatments were deemed necessary by the investigator, then it was ensured that a stable dose and therapy type were maintained throughout the study to minimize potential interference with the study endpoint assessments. Antipyretics on the day of injection were permitted, whereas all cancer treatments, with the exception of non-study-related local lesions palliative radiation therapy, were prohibited during the study.

### 2.4. Randomization and Blinding

Treatment allocation was performed prior to site initiation, and each patient was assigned a unique number based on the order of enrolment. Patients meeting the eligibility criteria were randomly assigned in a 2 : 1 ratio to either the THL-P treatment or placebo group. Randomization was achieved with the use of a permuted-block randomization algorithm with a block size of 6 in SAS 9.0 software (SAS Institute Inc., Cary, NC, USA), where a list of sequential numbers was generated with each number randomly assigned to a group. All patients, caregivers, investigators, and outcome assessors were blinded to treatment assignment.

### 2.5. Measurement of Outcomes

After randomization, several examinations were conducted at baseline and followed up every 4 weeks for 24 weeks. The examinations included self-administered EORTC-QLQ-C30, EORTC-QLC-Q30 and -Breast Cancer 23 (BR23), body weight, collection of blood for assessment of T-lymphocyte activity, biochemistry, and hematology tests.

The primary endpoint was to evaluate the efficacy of the treatment in improving the QOL of patients from baseline to last visit. The changes in the GHS/QOL scale were assessed by the self-administered EORTC-QLQ-C30. The secondary outcomes evaluated the efficacy and safety of the treatment. The efficacy of the treatment was evaluated between baseline and last visit via the following measures: (a) changes and maximum improvements in the functional and symptom standardized scales, as well as the single items, of the EORTC-QLC-Q30 and -Breast Cancer 23 (BR23), (b) changes in body weight, (c) immunomodulating effects of treatment on lymphocytes, as determined via phenotypic analyses of lymphocytes by flow cytometry (FACSCalibur, BD) with monoclonal antibodies for CD3, CD4, CD8, CD19, and CD16+56, and BD MultiSET software. The safety of the treatment was determined from the reports of adverse events.

### 2.6. Statistical Analyses

The sample size for this study was arbitrarily determined to be 60 subjects for this pilot study to collect useful information for further study in the future. Demographic and clinical characteristics at baseline were analyzed according to randomized treatment groups. Primary and secondary endpoints were analyzed based on the intent-to-treat (ITT) population, which was defined as all randomized patients that received any treatment. Data of continuous variables are presented as mean ± standard deviation. When the normality of these variables cannot be assumed, the data are presented as median (interquartile range). Data of categorical variables are presented as numbers (percentages). Statistical comparisons between continuous variables were made using the independent, two-sample *t*-test. The Mann-Whitney *U* test was used to compare independent groups of data that were not normally distributed. A Fisher's exact test was used for comparisons of categorical variables. A parametric Student's paired *t*-test or nonparametric Wilcoxon signed ranks test were used to compare differences before and after treatment in each group. For all analyses, a two-sided *P* value of <0.05 was considered significant. Our primary hypothesis is that there would be a significant improvement in the QOL between baseline and last-visit assessments in the THL-P versus placebo groups. Statistical analyses were performed using SPSS 15.0 statistics software (SPSS Inc, Chicago, IL, USA) and SAS 9.0 (SAS Institute Inc., Cary, NC, USA).

## 3. Results

### 3.1. Study Population

**Figure 1:**
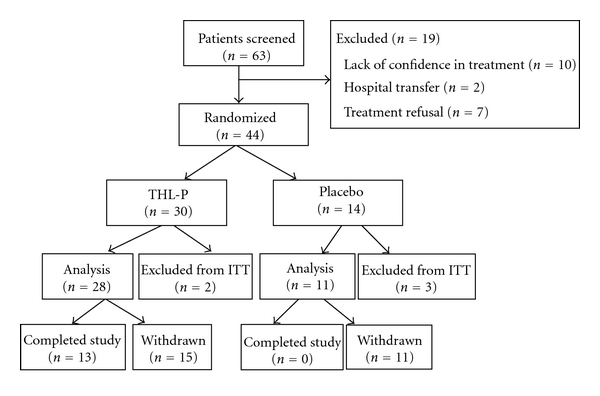
Flow diagram of the randomized, double-blind, placebo-controlled, parallel-group phase IIa clinical trial comparing Tien Hsien liquid practical (THL-P) to placebo for safety and efficacy. A total of 63 patients entered our trial between June 2009 and June 2011. Nineteen patients were excluded due to lack of confidence in the treatment (*n* = 10), hospital transfer (*n* = 2), and treatment refusal (*n* = 7). Forty-four patients were randomly assigned in a 2 : 1 ratio to either the THL-P group (*n* = 30) or the placebo group (*n* = 14). Thirteen patients in the THL-P group and none in the placebo group had completed the study.

A total of 63 patients entered our trial between June 2009 and June 2011, and 19 patients were excluded due to lack of confidence in the treatment (*n* = 10), hospital transfer (*n* = 2), and treatment refusal (*n* = 7). Overall, the screening failure rate was 30.2%, and 44 patients were randomly assigned in a 2 : 1 ratio to either the THL-P group (*n* = 30) or the placebo group (*n* = 14) (Figure 1). Of these 44 patients, 39 had metastasis in bone, 19 in liver, 18 in lung, and 4 in brain. The demographic and clinical characteristics of the 44 randomized patients are summarized in Table 1. There were no statistically significant differences between the THL-P and placebo groups in age, height, body weight, stage of cancer (i.e., according to the TNM classification), duration of onset, and the scales and single items of the EORTO-QLQ-C30. Of the patients that were randomized to treatment, 31 patients (70.5%) did not complete the study. Their withdrawal reasons were listed in Figure 1. In the THL-P group, 13 patients completed the study, while none of the patients in the placebo group completed the study. Interestingly, all of the participants in the placebo group withdrew prior to week 12, whereas only 11 participants (39.3%) in the THL-P group withdrew prior to week 12. The ITT population was comprised of 39 patients, with 28 patients in the THL-P group and 11 patients in the placebo group (Figure 1).

### 3.2. Efficacy

**Figure 2:**
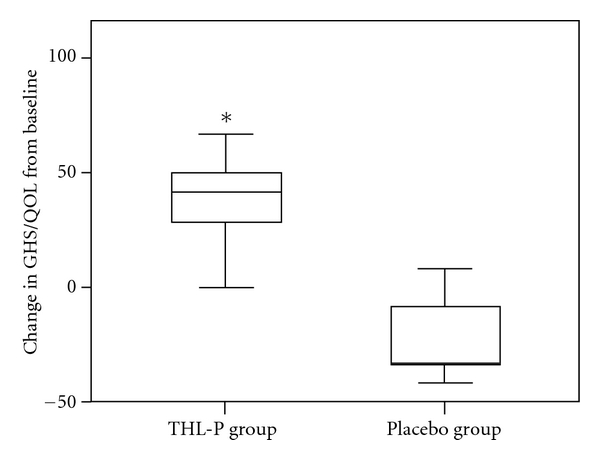
Primary endpoint of the intent-to-treat population. Changes in the standardized score of the global health/quality of life (GHS/QOL) scale of the European Organization for Research and Treatment of Cancer Quality of Life Questionnaire-Core 30 (EORTO-QLQ-C30) in the intent-to-treat population. Treatment of Cancer Quality of Life Questionnaire-Core 30 (EORTO-QLQ-C30). Values represent medians (interquartile ranges). *P < 0.05 versus placebo (Mann-Whitney *U* test).

The primary endpoint, in particular, changes in the GHS/QOL standardized scale between the baseline and last visit of the ITT population, is presented in Figure 2. There was a significant difference between the THL-P and placebo group with respect to the change in the GHS/QOL scale from the baseline and last visit (41.69 versus −33.33; *P* < 0.001, Table 2, Figure 2). These findings indicate that patients administered THL-P had a higher and improved QOL than those receiving a matched placebo. Thus, THL-P appears to be efficacious in improving the QOL of patients with refractory metastatic cancer.

Table 2 presents the primary endpoints for changes in the standardized functional and symptom scales, as well as the single items, of the EORTO-QLQ-C30. There were significant differences in the changes from baseline to last visit in four out of five functional scales (i.e., physical, role, emotional, and cognitive functions), as well as one symptom scale (i.e., fatigue), between the THL-P and placebo groups (*P* < 0.05). Indeed, the THL-P group appears to have improvements in these scales compared to the placebo group. There were no significant differences from baseline to last visit in the single items (i.e., dyspnoea, insomnia, loss of appetite, constipation, diarrhoea, and financial difficulties) of the EORTO-QLQ-C30 between the groups.

The secondary endpoints for changes in the standardized functional and symptom scales the EORTO-QLQ-BR23 are presented in Table 3. There was a significant decrease in the changes from baseline to last visit in the “systemic therapy side effects” scale between the THL-P and placebo groups (*P* < 0.05). Lastly, there were no significant differences in the body weights (i.e., % change) of the two groups between the baseline and last visit (Table 3).

### 3.3. Safety

The treatment-related adverse events reported during the study were mild constipation (*n* = 6) and localized itching (*n* = 1). Constipation was relieved by increasing water intake, and itching subsided immediately after cessation of all medication. No nausea, vomiting, hair loss, diarrhoea, and heavy constipation were observed. Nine severe adverse events were reported by five participants. However, all serious adverse events were considered unrelated to THL-P treatment by the IRB.

### 3.4. Immunomodulating Effects

There were significant differences in the changes from baseline to the last visit in CD3, CD4/CD8, CD19, and CD16+56 positive cells between the THL-P and placebo groups (*P* < 0.05) (Table 3). Specifically, compared with placebo, THL-P appears to have elevated the levels of CD3, CD4/CD8, CD19, and CD16+56 positive cells.

## 4. Discussion

In patients with refractory metastatic breast cancer, the oral administration of 20 mL THL-P three times a day for 24 weeks significantly improved the QOL, increased the physical, role, emotional, and cognitive functioning, decreased fatigue and systemic therapy side effects, and had immunomodulating effects on lymphocytes. Furthermore, THL-P treatment did not induce any severe adverse events, and the only side effects reported were mild constipation and localized itching. Together, these findings suggest that THL-P is a safe and effective CAM for patients with refractory metastatic breast cancer.

Currently, no global consensus or guidelines exist for the treatment of patients with metastatic breast cancer, especially for those refractory to conventional treatments [4, 5]. Furthermore, it is estimated that anywhere from 48 to 98% of all breast cancer patients use some form of CAM [10–13]. Interestingly, achieving the best quality of life (QOL) is one of the main goals of conventional treatments, as well as reasons for using CAM [4, 5, 10, 12, 13]. It was recently reported that the GHS/QOL scale, assessed via the EORTC-QLQ-C30, can serve as an important predictor of response to treatment, PFS, and OS in women with metastatic breast cancer [16]. In the present study, which used the same questionnaire to assess the GHS/QOL, as well as the function and symptom scales, found that THL-P was effective in improving the QOL and functions of refractory metastatic breast cancer patients. Given these positive findings, larger trials examining the effects of THL-P on other pertinent outcomes, such as PFS and OS, are warranted.

The use of CAM has been on the rise worldwide, especially among patients with life-threatening diseases, such as cancer [9]. There are also marked cultural differences in the way CAM is integrated into breast cancer treatment regiments [13]. In Asian populations, including Taiwan, the use of traditional Chinese medicines is very common and enjoys widespread popularity [13, 17]. However, the use of CAM may be associated with severe adverse effects or CAM-drug interactions with conventional treatments for breast cancer, and consequently their safety and efficacy warrant investigation via randomized and controlled clinical trials [13, 18]. In this randomized, double-blind, placebo-controlled, parallel-group, phase IIa trial, it was found that THL-P was not only an effective CAM in patients with refractory metastatic breast cancer, but also a safe alternative adjuvant, since there were no serious adverse events reported with its use. Although these findings suggest that the use of THL-P is safe and effective, studies that examine the safety of using THL-P, as well as other CAMs, in combination with conventional treatments are needed.

In the present study, there were more withdrawal cases in the placebo group than in the THL-P treatment group. Given that this was a double-blinded study, where the patients, caregivers, investigators, and outcome assessors were blinded to treatment, we speculate that the significant difference in the number of withdrawal cases between the control and study groups suggests that THL-P may have possible therapeutic effects in patients with refractory metastatic breast cancer, which requires further investigation.

Although not directly studied, the therapeutic efficacy of THL-P in patients with metastatic breast cancer can be extended to its antimetastatic, antiangiogenic, and antitumour effects, as previously demonstrated via *in vitro *and *in vivo* preclinical studies [6]. Furthermore, corroborating the observations of the present study, THL-P was also found not to have any adverse effects on the body weights of immunocompromised mice [6]. In addition to its anticancer effects, THL-P was found to have immunomodulating effects, specifically in reducing cytokine production of T-lymphocytes isolated from patients with recurrent aphthous ulcerations [14, 15]. Herein, we also demonstrated that THL-P has immunomodulating effects in patients with refractory metastatic breast cancer. Specifically, we found that there were marked differences in the changes from baseline to the last visit in CD3, CD4/CD8, CD19, and CD16+56 positive cells between the THL-P and placebo groups. Although our study provided the first clinical evidence for the safety and efficacy of using THL-P in refractory metastatic breast cancer patients, future studies assessing the specific mechanisms responsible for its immunomodulating effects in this population are warranted.

CAMs are widely used by breast cancer patients; however their safety and efficacy have been studied in very few randomized and controlled clinical trials [8, 13, 19]. The present study is one of a few to provide safety and efficacy evidence for a CAM that was beyond empirical evidence, case studies, and hypothetical physiological effects. Despite these important findings, there are several limitations in the present study, including that this was a single-site study with a small sample size and that THL-P is a compound with multiple ingredients. Therefore, further investigations on the exact therapeutic mechanisms for each ingredient of THL-P, as well as multiple site-, large-scale studies that confirm and extend the safety and efficacy findings of the present study, are warranted. However, it should be mentioned that, to our best knowledge, there are no studies reporting that the active ingredients of THL-P have any estrogenic effects or pose a risk for increased tumour growth [20–30].

## 5. Conclusions

Our findings suggest that an oral administration of 20 mL THL-P three times a day for 24 weeks significantly improves the QOL, increases the physical, role, emotional, and cognitive functioning, decreases fatigue and systemic therapy side effects, and has immunomodulating effects on lymphocytes in patients with refractory metastatic breast cancer. Additionally, THL-P treatment did not induce any severe adverse events. Together, these findings suggest that THL-P is a safe and effective CAM for patients with refractory metastatic breast cancer.

## Figures and Tables

**Table 1:** Demographic and clinical characteristics of patients with refractory metastatic breast cancer at baseline.

	THL-P (*n* = 30)	Placebo (*n* = 14)	*P* value
Demographics			

Age (years)^a^	60.7 ± 9.5	58.7 ± 7.6	0.507
Height (cm)^a^	155.3 ± 5.9	156.5 ± 5.4	0.512
Weight (kg)^a^	57.6 ± 8.1	59.3 ± 10.5	0.554
T stage, *n* (%)^b^			
T1	4 (13.4)	1 (7.2)	0.968
T2	12 (40.0)	7 (50.0)	
T3	7 (23.3)	3 (21.4)	
T4	7 (23.3)	3 (21.4)	
Lymph nodes, *n* (%)^b^			
N0	10 (33.3)	3 (12.4)	0.306
N1	6 (20.0)	2 (14.3)	
N2	4 (13.4)	2 (14.3)	
N3	10 (33.3)	7 (50.0)	
Metastasis, *n* (%)^b^			
M1	30 (100)	14 (100)	N/A
Duration of onset (years)^c^	3.5 (2.0, 7.0)	4.1 (2.7, 5.4)	0.970

EORTO-QLQ-C30			

*GHS/QOL* ^ c^	37.5 (16.7, 50.0)	50.0 (25.0, 66.7)	0.082
*Functional scales*			
Physical^c^	60.0 (46.7, 73.3)	70.0 (46.7, 86.7)	0.336
Role^c^	66.7 (33.3, 83.3)	66.7 (33.3, 100.0)	0.643
Emotional^c^	66.7 (50.0, 83.3)	75. 0 (58.3, 91.7)	0.469
Cognitive^c^	66.7 (50.0, 83.3)	83.3 (66.7, 83.3)	0.118
Social^c^	66.7 (33.3, 100.0)	66.7 (66.7, 100.0)	0.711
*Symptom scales*			
Fatigue^c^	55.6 (33.3, 66.7)	38.9 (22.2, 55.6)	0.273
Nausea and vomiting^c^	0 (0, 16.7)	0 (0, 0)	0.362
Pain^c^	33.3 (0, 66.7)	8.3 (0, 50.0)	0.382
*Single items*			
Dyspnoea^c^	33.3 (0, 66.7)	0 (0, 33.3)	0.069
Insomnia^c^	33.3 (0, 33.3)	33.3 (0, 66.7)	0.608
Appetite loss^c^	33.3 (0, 66.7)	0 (0, 33.3)	0.367
Constipation^c^	0 (0, 33.3)	0 (0, 33.3)	0.403
Diarrhoea^c^	0 (0, 0)	0 (0, 33.3)	0.523
Financial difficulties^c^	33.3 (0, 33.3)	33.3 (0, 33.3)	0.746

THL-P: Tien-Hsien liquid practical: EORTO-QLQ-C30: European Organization for Research and Treatment of Cancer Quality of Life Questionnaire-Core 30; GHS: global health status; QOL: quality of life.

*P* values were determined via the independent, two sample *t*-test^a^, Fisher's exact test^b^, and Mann-Whitney *U *test^c^. Values are presented as mean ± standard deviation^a^, number (percentage)^b^, and median (interquartile)^c^.

**Table 2:** Primary endpoints: Changes in the functional and symptom scales of EORTO-QLQ-C30 in the intent-to-treat population.

	THL-P (*n* = 28)	Placebo (*n* = 11)	*P*-value
*Change from baseline*			
EORTO-QLQ-C30			
*GHS/QOL*	41.7 (29.2, 50.0)^†^	−33.3 (−33.3, 0)^†^	<0.001*
*Functional scales*			
Physical	13.3 (3.3, 26.7)^†^	0 (−13.3, 13.3)	0.014*
Role	0 (0, 41.7)^†^	0 (−16.7, 0)	0.018*
Emotional	8.3 (0, 25.0)^†^	0 (−33.3, 8.3)	0.024*
Cognitive	16.7 (0, 16.7)^†^	0 (−33.3, 0)	<0.001*
Social	0 (0, 33.3)^†^	0 (−33.3, 33.3)	0.379
*Symptom scales*			
Fatigue	−22.2 (−33.3, −11.1)^ †^	0 (−11.1, 22.2)	<0.005*
Nausea and vomiting	0 (0, 0)	0 (0, 16.7)	0.656
Pain	0 (−25.0, 16.7)	0 (0, 16.7)	0.124
*Single items*			
Dyspnoea	0 (−33.3, 0)	0 (0, 0)	0.528
Insomnia	0 (0, 16.7)	0 (0, 33.3)	0.569
Appetite loss	0 (0, 0)	0 (0, 0)	0.633
Constipation	0 (−16.7, 0)	0 (0, 0)	0.770
Diarrhoea	0 (0, 33.3)	0 (0, 33.3)	0.866
Financial difficulties	0 (0, 0)	0 (0, 0)	0.747

THL-P: Tien-Hsien liquid practical; EORTO-QLQ-C30: European Organization for Research and Treatment of Cancer Quality of Life Questionnaire-Core 30.

*P < 0.05 versus placebo (Mann-Whitney *U *test). ^†^ *P* < 0.05 versus baseline (Wilcoxon signed ranks test). Values are presented as median (interquartile).

**Table 3:** Secondary endpoints: changes in the functional and symptom scales of EORTO-QLQ-BR23, lymphocytes, and body weight in the intent-to-treat population.

	THL-P (*n* = 28)	Placebo (*n* = 11)	*P* value
*Change from baseline*			
EORTO-QLQ-BR23			
*Functional scales* ^ a^			
Body image	0 (0, 33.3)^†^	0 (0, 8.3)	0.346
Sexual function	0 (0, 0)	0 (0, 0)	0.591
Sexual enjoyment	0 (0, 0)	16.7 (0, 33.3)	0.582
Future perspective	16.7 (0, 33.3)^†^	0 (0, 0)	0.102
*Symptom scales* ^ a^			
Systemic therapy side effects	−4.8 (−23.8, 0)^†^	4.8 (−4.8, 9.5)	0.010*
Breast symptoms	0 (−16.7, 8.3)	0 (−8.3, 0)	0.450
Arm symptoms	−5.6 (−33.3, 0)^ †^	0 (−11.1, 22.2)	0.346
Upset by hair loss	−33.3 (−33.3, 0)^ †^	0 (−16.7, 0)	0.316
Lymphocytes (%)			
CD3	6.0 (1.0, 9.5)^†^	−2.5 (−6.0, −1.0)	0.001*
CD4	0.0 (−2.0, 8.0)	−1.5 (−4.0, 1)	0.157
CD8	−1.5 (−3.0, 1.5)	0.5 (−2.0, 2.0)	0.387
CD4/CD8	0.2 (−0.1, 0.5)^†^	−0.1 (−0.2, 0.0)	0.043*
CD19	3.0 (0.0, 7.5)^†^	−2.0 (−3.0, 3.0)	0.021*
CD16+56	8.0 (3.0, 11.0)^†^	−4.0 (−6.0, −3.0)^†^	<0.001*
Body weight (%)^b^	0.2 ± 3.4	0.3 ± 4.0	0.964

THL-P: Tien Hsien liquid practical; EORTO-QLQ-BR23: European Organization for Research and Treatment of Breast Cancer-Specific Quality of Life Questionnaire.

*P < 0.05 versus placebo (Mann-Whitney *U *test^a^ or independent, two-sample test^b^).

^†^ *P* < 0.05 versus baseline (Wilcoxon signed ranks test^a^ or paired Student's *t*-test^b^). Values are presented as median (interquartile)^a^ and mean ± standard deviation^b^.
